# Numerical Analysis on the Thermal Performance in an Excavating Roadway with Auxiliary Ventilation System

**DOI:** 10.3390/ijerph18031184

**Published:** 2021-01-29

**Authors:** Zijun Li, Huasen Liu, Yu Xu, Rongrong Li, Mintao Jia, Mengsheng Zhang

**Affiliations:** 1 School of Resources and Safety Engineering, Central South University, Changsha 410083, Hunan, China; zijunli@csu.edu.cn (Z.L.); liuhs@csu.edu.cn (H.L.); rongrongli@csu.edu.cn (R.L.); jmt321@163.com (M.J.); zms1998520@163.com (M.Z.); 2 Sinosteel Maanshan General Institute of Mining Research Co., Ltd., Maanshan 243000, Anhui, China

**Keywords:** heat hazard, roadway construction, deep underground, working face, orthogonal test

## Abstract

A steady and proper thermal environment in deep underground is imperative to ensure worker health and production safety. Understanding the thermal performance in the roadway is the premise of temperature prediction; ventilation design; and improvement in cooling efficiency. A full coupled model incorporated with a moving mesh method was adopted; reflecting the dynamic condition of roadway construction. This study revealed the characteristics of the thermal performance and its evolution law in an excavating roadway. Several scenarios were performed to examine the designs of the auxiliary ventilation system on thermal performance in the roadway. The results show that there is a limitation in the cooling effect by continuously increasing the ventilation volume. Reducing the diameter of the air duct or distances between the duct outlet and the working face will aggravate the heat hazard in the roadway. The heat release from the roadway wall increases with the increase of the advance rate of the working face or roadway section size. Furthermore; an orthogonal experiment was conducted to investigate the effect of major factors on the average air temperature and local heat accumulation in the roadway

## 1. Introduction

With the increase in decreasing of shallow resources, deeper resource exploitation in high-temperature circumstances has become a necessity [1,2]. The thermal stresses resulted from high-temperature surrounding rock become a major challenge for the deep construction and safe operation [3,4]. The high-temperature environment not only threatens the health of workers, reduces the work efficiency, but also shortens the mechanical lifetime limit [5,6,7]. The heat hazard occurs frequently in the development zone of roadway owing to the high original rock temperature and poor ventilation [8]. The auxiliary ventilation is the major and common method for providing cooling energy and controlling heat hazards in the construction of roadway [9,10]. It is a challenge that designing an appropriate ventilation system and improving the cooling performance to control heat hazard in the high temperature roadway. 

The mechanical ventilation is a crucial factor to eliminate pollutants and ensure safe production in the roadway. Therefore, a series of studies have been conducted by scholars to design an appropriate ventilation pattern aiming at different mining environment. Parra et al. established three types of ventilation models to identify the dead zone and the risk of methane explosion under different criteria respectively [11]. Hasheminasab et al. examined the distribution of methane in the development zones of underground coal mines at different ventilation scenarios [12]. Chang et al. [13] and Huang et al. [14] analyzed the dispersion and accumulation of CO in the roadway and determined areas with lower discharge. Wang et al. performed a long roadway model and revealed the relationships among the pressure difference, the air leakage rate and air quantity [15]. The airflow conditions have a significant relationship with dust movement, the extensive studies also have been carried out to examine the diffusion rules of dust and the optimal dust-removal airflow rate [16,17,18]. These subjects have been widely studied, but there are few studies focused on the problem of heat hazard control by auxiliary ventilation in the mine roadway, and the auxiliary ventilation plays an important role in regulating air temperature [19].

The thermal performance in the roadway can be classified into two types: operation period and excavated period. In an operation roadway, the airflow velocity near the wall of the roadway is small, and the heat transfer is relatively stable and slow [20,21]. The local temperature difference in airflow is low. The fluctuation of airflow temperature inside the roadway is mainly determined by the ventilation temperature, the length of the roadway and ventilation time [22,23]. While in an excavating roadway, the temperature of airflow and surrounding rock change more dramatically and rapidly [24,25]. Especially in the development zone, the airflow is a turbulent state, and there are significant differences in airflow velocity in the wall of roadway, which results in obvious differences in heat flux of the surrounding rock [26]. 

Maintaining a steady and comfortable thermal environment in underground spaces has received more attention in recent years due to the more severe heat hazard situation. Various cooling systems such as split-type vapor compression refrigerators [27], high temperature exchange machinery system (HEMS) [28,29], liquid carbon dioxide cycle refrigeration systems [30] et al. have been proposed for removing heat and improving working conditions. Although these approaches can provide a huge amount of cryogenic energy for heat hazard control in the underground, an improper application of cold air will increase the cost of cooling energy and reduce the thermal comfort for workers. Therefore, understanding the cooling characteristic of air temperature in the roadway and the influence of ventilation pattern is absolutely critical for heat hazard control.

Several studies investigated the characteristics of airflow and heat transfer in the development zones to predict the thermal performance and design the schemes of heat hazard control. The thermal performance in the roadway is affected by variety of factors, such as the temperature of rock and ventilation, state of airflow, mining situation, production time, et al. [31,32] Zhang et al. developed a physical simulation test system and explored the evolution law of temperature for surrounding rock [33]. Wang et al. calculated the heat release source from the roadway based on ignoring the local temperature difference, and proposed the prediction formula for airflow temperature in the roadway [34]. Besides, some of numerical models have been developed to describe the heat transfer in rock mass [35,36,37]. Habibi et al. measured the rock thermal conductivity and developed a numerical model investigating the heat transfer between air and rock [38]. In addition, Ji et al. theoretically analyzed the characteristics of heat transfer at working face under a jet flow [39].

Although a considerable quantity of studies focused on the cooling system and heat transfer in a roadway, few research revealed the thermal performance in an excavating roadway, considering the convective heat transfer between surrounding rock and airflow, unsteady-state heat transfer in rock, non-isothermal flow in the roadway, and the advance of the working face. To reflect the dynamic condition of roadway construction, a fully coupled mathematical model incorporated with a moving mesh method is adopted. The aim is to understand the cause of high temperature in the excavating roadway and efficiently control the heat damage. The characteristics of the thermal performance in an excavating roadway, and its evolution law are generally obtained and analyzed. The crucial factors such as the ventilation volume, the diameter of air duct, the distance between duct outlet and working face and the roadway section size affecting the air temperature in the roadway will be investigated in detail. An orthogonal test is carried out to comprehensively analyze the experiment and find out the influence rule of each factor on the thermal performance in the excavating roadway. This study can provide a robust theoretical basis for saving cooling energy in heat hazard control and improving the thermal comfort in roadway construction.

## 2. Numerical Methods

In construction of a roadway, the heat hazard is most severe in the development zones as the high-temperature of the original rock. The thermal performance inside the roadway is affected by the airflow and temperature of the rock, which is a fluid-solid coupling process. The airflow state is crucial to the heat transfer in the wall of roadway, which is determined by solving the continuity, momentum, and turbulence model based on the Navier–Stokes equation. The realizable k–ε turbulent model is selected as the governing equation for the turbulent kinetic energy, and it has great advantages in simulation for isothermal flow, especially near-wall flow it has good accuracy and efficiency [40,41]. The energy balance equation is used to control the heat transfer in rock and fluid.

**Figure 1:**
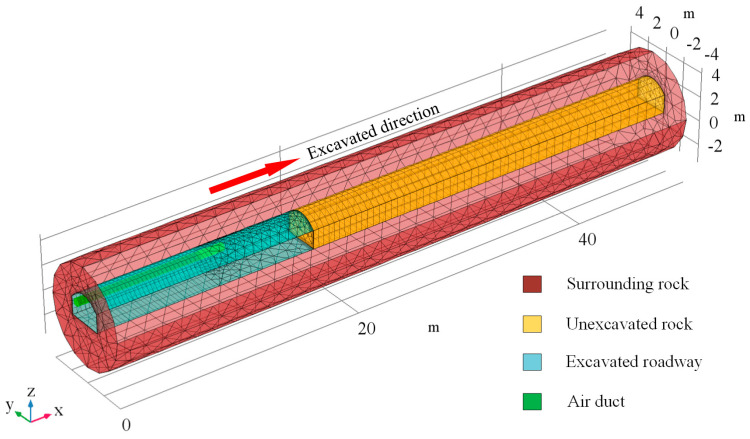
The initial established grid of the physical model.

The established geometric model is shown in Figure 1, and it is similar to that of the actual situation, including an excavating roadway equipped with an air duct, unexcavated rock and surrounding rock. The shape of the roadway section adopts the conventional arch roadway with a width of 5 m, a straight wall of 1.5 m, an arch height of 0.75 m. The initial length of the excavated roadway is 18 m, as well as the length of the air duct is 12 m. The auxiliary ventilation duct with a diameter of 0.6 m is arranged on the left side of the roadway, and the duct outlet is 6 m away from the working face. The original temperature of the surrounding rock and air in the roadway is 45 °C, and the advance rate of the working face is 0.2 m/h. The main parameters of the model are set in Table 1, which are substituted from the contemporary literature [42,43].

**Figure 2:**
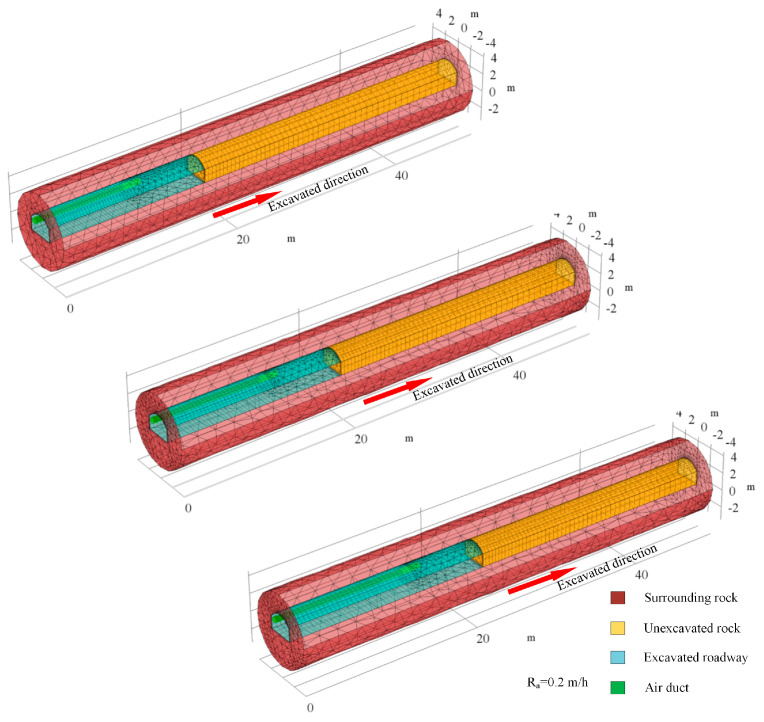
The moving meshes at different time.

A moving mesh method is incorporated into the mathematical model to perform the dynamic excavation of the roadway. The advance of the working face represents the excavation of the roadway. In the case that the region of roadway and rock are transformed as the advance of working face, the mapped mesh will deform with the movement of the boundary. Avoiding the mesh distortion arisen from the deformation of mapped mesh, the Laplace smoothing method is adopted to modify the deformation of the mesh by global convergence. Figure 2 depicts the grid movement of the physical model. In the process of roadway excavation, the working face of the roadway gradually advances to the unexcavated rock, and the length of the roadway increases correspondingly. The distance between the duct outlet and the working face mains the same. All the governing equations are implemented and numerically solved using the COMSOL Multiphysics software based on the finite element method. Simultaneously, as the temperature of surrounding rock and heat transfer are related to time, the transient solver is selected.

## 3. Numerical Results

### 3.1. Numerical Simulation of the Airflow Field

**Figure 3:**
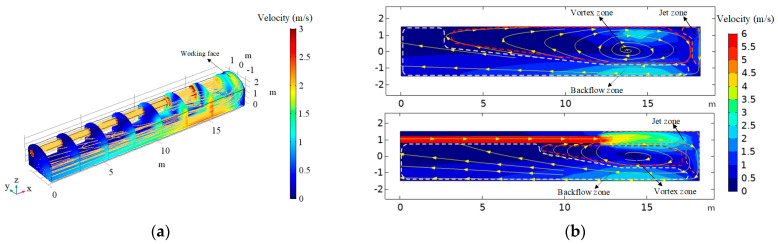
Air flow velocity distribution in the roadway: (**a**) air velocity isoclines in the *Y* direction; (**b**) airflow field in the different *Z* plane.

The characteristic of airflow in the development zone of the roadway during the excavation is shown in Figure 3. The condition of the airflow state near the working face is very complicated. The cold fresh air is released from the air duct to the roadway, and most of it impinges on the working face and then turns and flows to the roadway outlet. Near the working face, the airflow velocity on the two sides of the walls is larger. Three airflow fields can be distinguished in the roadway: Jet zone, Backflow zone, and Vortex zone. The Jet zone is located in the space between the duct outlet and the working face, the airflow jetted from the air duct to the working face, and the air along the path is continuously sucked in, which leads to an increase and diffusion of air along the path. As the limitation of space, the airflow collided with the left-hand side wall of the roadway and the working face generates an opposite direction airflow in the Backflow zone (right-hand side wall), and then it flows toward the roadway outlet. A swirling vortex flow formed in the middle of the roadway near the working face and exhibited a triangle pattern, and the reason for the formation is the entrainment action of the jet airflow in Jet zone and return airflow in the Backflow zone. The velocity of airflow is the highest in the jet region and the lowest in the vortex region.

### 3.2. Numerical Simulation of the Temperature Field

**Figure 4:**
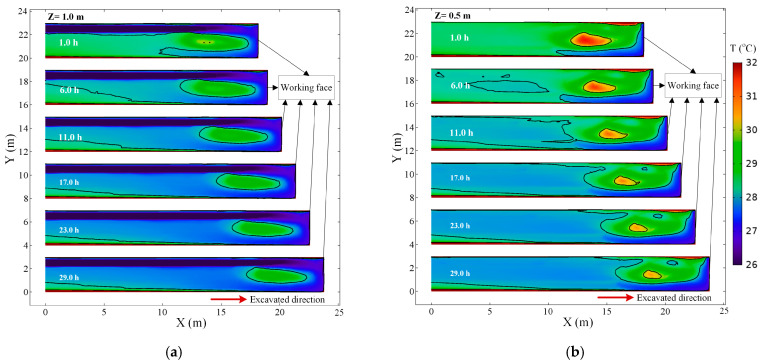
Air temperature distribution in the roadway at different times: (**a**) air temperature field in the *Z* = 0.5 m plane; (**b**) air temperature field in the *Z* = 1 m plane.

The cold fresh air supplied by auxiliary ventilation flows in the roadway, causing simultaneous cooling of the surrounding rock and heating of the airflow due to convective heat transfer. The evolution of the temperature field in the development of roadway is evident in Figure 4. At the beginning stages of auxiliary ventilation, the temperature decreases signally in the most zone of the roadway, and a local high-temperature region is presented. Compared Figure 4 with the airflow in Figure 3, it can be found that the temperature characteristics in the roadway are associated with the airflow state. The lowest temperature region is located in the Jet zone due to the cold fresh air supplied by auxiliary ventilation. In the Backflow Zone, the temperature of airflow increases significantly, especially in the area near the wall. It is evident that the heat transfer between the airflow and the roadway wall is larger here. When the airflow flows toward the roadway outlet, only a small part of the airflow is sucked into the Vortex zone. Consequently, the air in the Vortex zone is hard to refresh, and its temperature is higher than in the surrounding region. The local high-temperature zone moves forward as the advance of the working face and keeps a constant distance (4–5 m) from the working face. 

**Figure 5:**
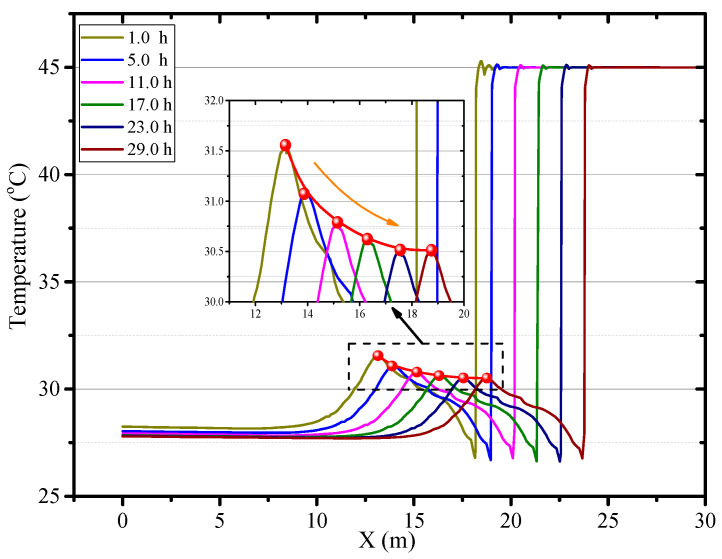
Air temperature in central axis of roadway (*Z* = 0.5) at different times.

With the increase of ventilation time, the surrounding rock is continuously cooled, and the heat exchange between the airflow and the wall of the roadway decreased, so the air temperature in the roadway decreased. As the ventilation progresses, the heat transfer between the rock and airflow achieved a thermal equilibrium, and the temperature field in the roadway tends to stabilize. Figure 5 presents the air temperature in the central axis of the roadway (Z = 0.5 m) at different times. The peak of air temperature does not exist near the working face. On the contrary, the temperature near the working face is low, and the air temperature within 1 m from the working face is between 27.8–28.2 °C. The air temperature in the roadway rises first and then decreases with the increase of distance from the working face. The air temperature peak existed 5 m away from the working face. After 1 h of ventilation, the air temperature peak on the curve is 31.6 °C, and 3.3 °C higher than the air temperature at the exit of the roadway. The air temperature peak gradually decreased with the ventilation time, and finally stabilized at about 30.5 °C. The air temperature at the exit of the roadway decreased from 28.2 °C in the 1 h to 27.7 °C in the 29 h. 

## 4. Sensitivity Analysis for Single Factor

There is no doubt that the air temperature in roadway will be directly affected by the auxiliary ventilation system and mining situation. So a number of scenarios are simulated to examine the thermal performance under different auxiliary ventilation pattern and excavated condition.

### 4.1. The Effect of Ventilation Volume

**Figure 6:**
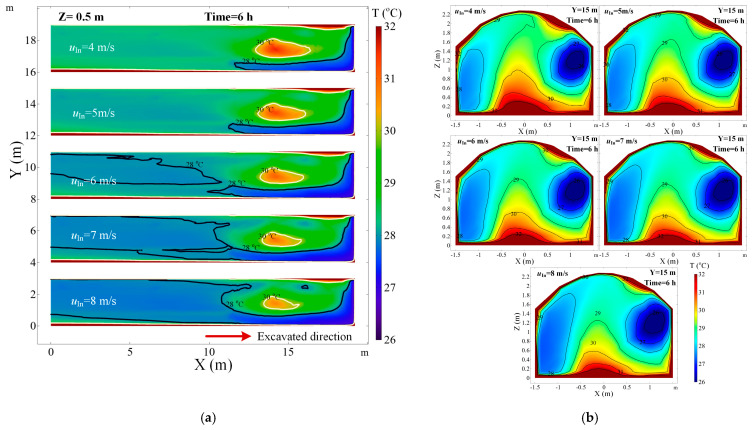
Air temperature distribution in roadway under different ventilation velocity: (**a**) air temperature in the *Z* = 0.5 m plane; (**b**) air temperature in the *Y* = 15 m section.

**Figure 7:**
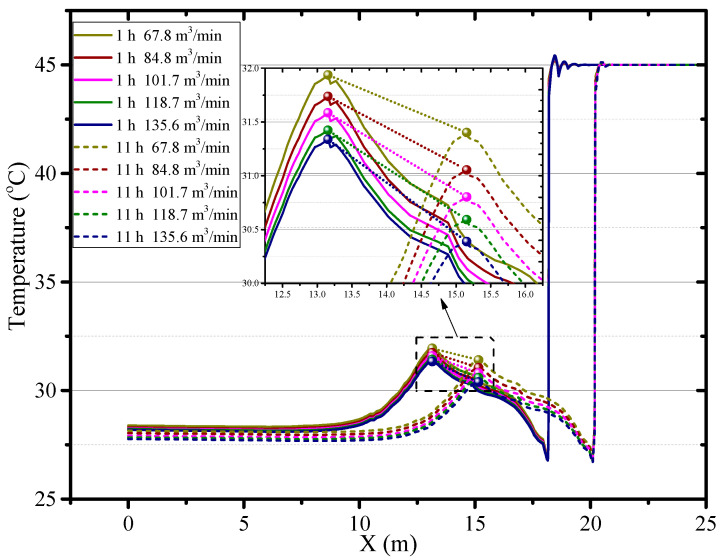
Air temperature in central axis of roadway (*Z* = 0.5 m) for different times.

The ventilation volume had a greater impact on the airflow field and the convective heat transfer between airflow and surrounding rock, so it is an essential factor for the thermal performance in the roadway. Five cases with different air duct ventilation velocity are performed in this section, i.e., u = 4, 5, 6, 7 and 8 m/s, and the other parameters, *D_s_* = 6 m, *d_a_* = 0.6 m, *T_r_* = 45 °C, *R_a_* = 0.2 m/h, remain unchanged. Figure 6 depicts the air temperature distribution in the roadway under different ventilation velocity. A local high-temperature zone still existed in the middle of the roadway regardless of the ventilation volume, and it expands with decreasing ventilation volume. The ventilation volume has an obvious influence on the temperature at the place near the working face and the right-hand wall of the roadway, where the air temperature decreases significantly with increasing ventilation volume. Figure 7 shows that the air temperature in the central axis of roadway decreases with the increase of ventilation volume, and the peak of air temperature decreases more quickly as the ventilation time. The larger the ventilation volume decreases the air temperature in most zone of the roadway, especially in high airflow velocity areas as the quick heat diffuses. Compared to the curves in Figure 7, the air temperature difference between the curves is gradually reduced with increases in ventilation volume, so there is a limit to adjust the air temperature in roadway by increasing ventilation volume.

### 4.2. The Effects of the Diameter of Air Duct

**Figure 8:**
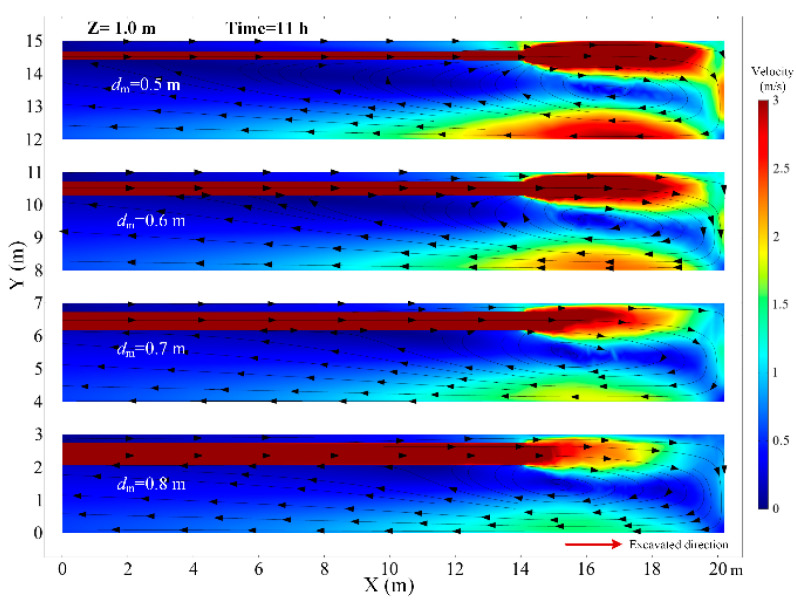
Airflow velocity distribution in *Z* = 1.0 m plane under different diameter of air duct.

**Figure 9:**
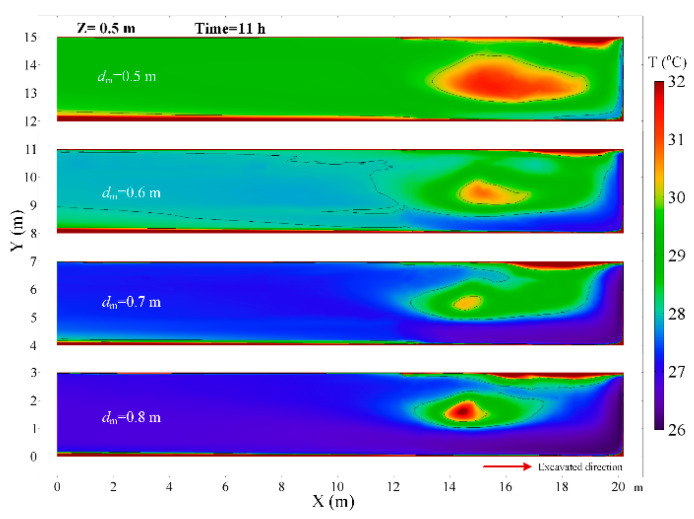
Air temperature distribution in *Z* = 0.5 m plane under different diameter of air duct.

**Figure 10:**
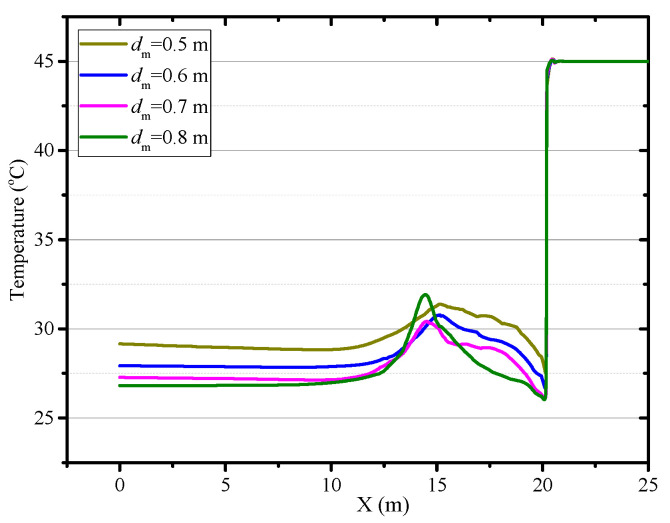
Air temperature in central axis of roadway (*Z* = 0.5 m) under different diameter of air duct.

The diameter of the air duct directly affects the released airflow velocity, so it is a critical factor to the thermal performance in the roadway. In this section, the airflow and thermal performance in the roadway with a constant ventilation volume of Q = 102 m^3^/min and diameter of air duct sizes of *d_m_* = 0.5, *d_m_* = 0.6, *d_m_* = 0.7 and *d_m_* = 0.8 m are investigated. Figure 8 shows that when the diameter of the air duct decreases, the airflow velocity increases in the roadway especially near the roadway wall and the working face, which increases the convective heat transfer between the surrounding rock and airflow. Therefore, the smaller the air duct, the higher the air temperature in the roadway and the larger the area of the local high-temperature zone, as shown in Figure 9. When the diameters of the air duct are 0.5, 0.6, 0.7 and 0.8 m respectively, the air temperatures in the exit of the roadway are 29.16 °C, 27.93 °C, 27.28 °C, 26.81 °C respectively, the maximum difference in the air temperatures is 2.35 °C. When the diameter of the air duct is 0.8 m, the maximum temperature in the vortex zone is higher than that when the diameter of the air duct is 0.7 m. This is because although the reduction in duct diameter reduces the area of the vortex zone, the low airflow velocity results in a more serious accumulation of heat in the vortex zone. Figure 10 shows that there is an obvious variation in air temperature near the working face as the air duct diameter, which results from the change in airflow velocity. In general, increasing the diameter of the air duct has a benefit to improving the cooling effect in the roadway by weakening convection heat transfer in the working face. However, the selection of air duct diameter is limited by the roadway construction space.

### 4.3. The Effect of the Distance between Duct Outlet and Working Face

**Figure 11:**
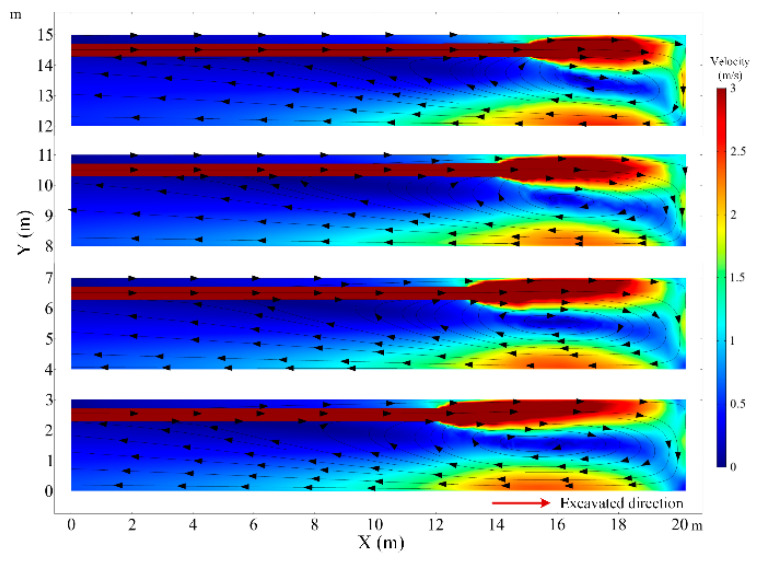
Airflow velocity distribution in *Z* = 1.0 m plane under different distance between duct outlet and working face.

**Figure 12:**
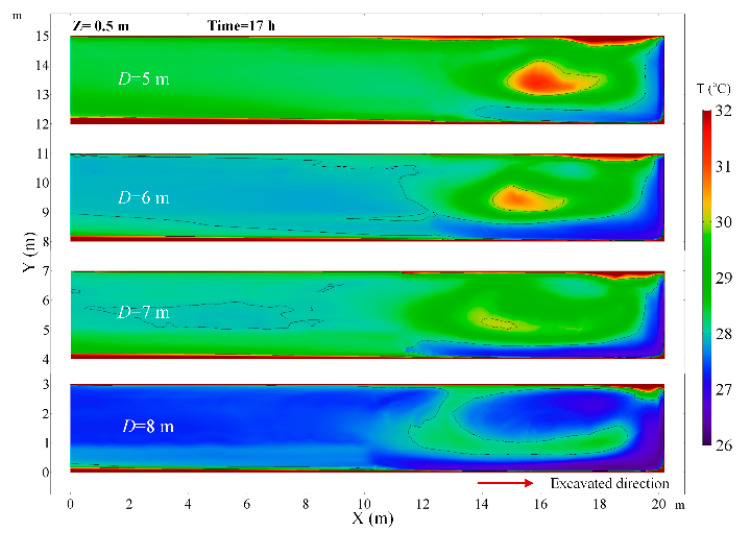
Air temperature distribution in *Z* = 0.5 m plane under different distance between duct outlet and working face.

**Figure 13:**
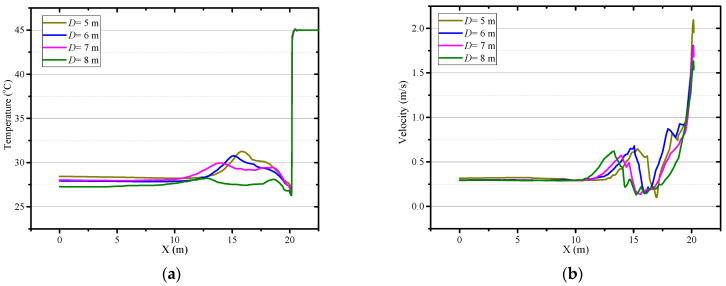
Airflow velocity (**a**) and temperature (**b**) in central axis of roadway (Z = 0.5 m) under different distance be-tween duct outlet and working face.

Figure 11 depicts the airflow performance with a constant ventilation volume of Q = 102 m^3^/min and different distances between the duct outlet and working face. Increasing the distance between the duct outlet and the working face, the center of the vortex zone is further away from the working face. Meanwhile, the area of the local high-temperature zone reduces with the increase of distance between the duct outlet and working face, as shown in Figure 12. So the increase of the distance between the duct outlet and working face can prevent the accumulation of heat in the roadway to some extent. As we can see from Figure 13a, when the distance between the duct outlet and working face is 8 m, there is no obvious peak of air temperature on the center line. In general, the longer the distance between the duct outlet and working face, the lower the air temperature in the roadway. According to Figure 13b, it can be concluded that increasing the distance between the air duct and the working face will reduce the airflow velocity near the working face, so where the convective heat transfer between the surrounding rock and airflow decreases, which results in a reduction of heat release from the working face. Therefore, the cooling effect in the roadway can be appropriately enhanced by increasing the distance between the air duct and the working face. Nonetheless, it should pay attention to the accumulation of toxic and harmful gases near the working face caused by the reduction of airflow near the working face as the increase of the distance between the air duct and the working face.

### 4.4. The Effects of the Advance Rate of the Working Face

**Figure 14:**
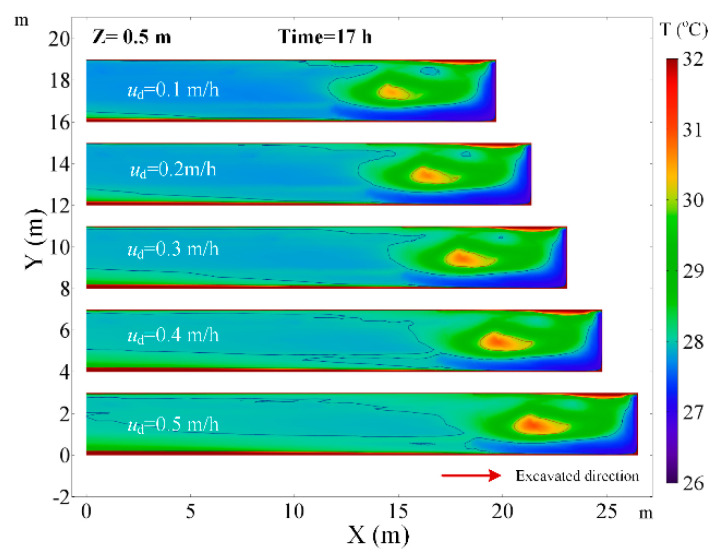
Air temperature distribution in *Z* = 0.5 m plane under different advance rate of the working face.

**Figure 15:**
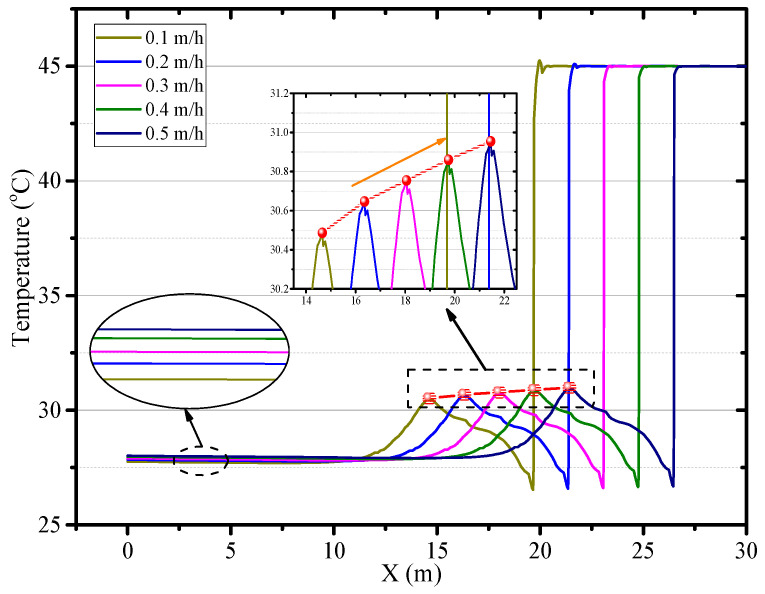
Air temperature in central axis of roadway (*Z* = 0.5 m) under different advance rate of the working face.

Figure 14 depicts that the air temperature in the Z = 0.5 m plane under a different advance rate of the working face. The adjustment of the advance rate of the working face has no significant effect on the airflow field. At a different advance rate of the working face, the local high-temperature zone maintains the same distance from the working face and the air temperature in the local high-temperature zone is higher. The air temperature in the central axis of roadway increases with the mining speed as shown in Figure 15. As the advance rate of the working face is increased from 0.1 to 0.5 m/s, the peak of air temperature on the central axis of roadway raises from 30.48 to 30.96 °C. The main reason is that the faster the advance rate of the working face is, the higher the temperature of the working face, and more heat release from the working face. Besides, the rapid growth of the length of roadway also increases the heat release from the surrounding rock. Therefore, decreasing the advance rate of the working face can improve the thermal environment for the workers.

### 4.5. The Effects of the Roadway Section Size

**Figure 16:**
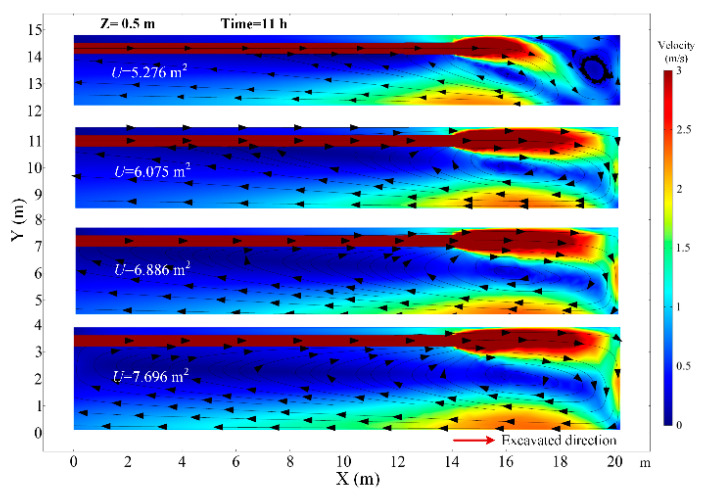
Airflow velocity distribution in *Z* = 1.0 m plane under different roadway section size.

**Figure 17:**
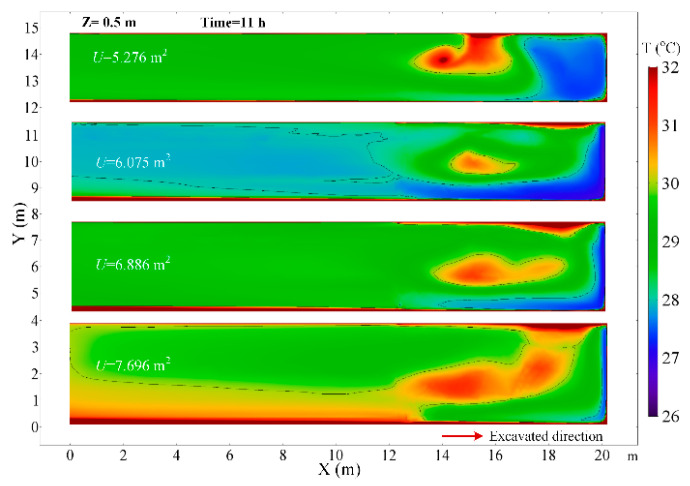
Air temperature distribution in *Z* = 0.5 m plane under different roadway section size.

**Figure 18:**
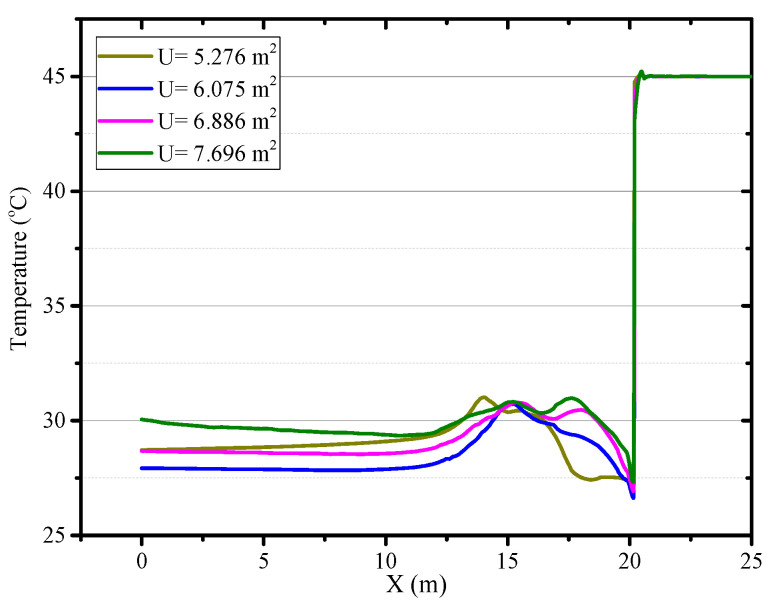
Air temperature in central axis of roadway (*Z* = 0.5 m) under different roadway section size.

At a constant ventilation volume, the variation of the size of the roadway section will affect the movement of airflow in the roadway. The thermal performance in the roadway under different roadway section sizes of U = 5.276, U = 6.075, U = 6.886 and U = 7.696 m^2^ are investigated. When the roadway section size is 5.276 m^2^, the airflow field has an obvious change in the roadway and there are two cycle flow areas in the Z section as shown in Figure 16. The airflow jetted from the air duct does not flow directly along the wall to the working face, but first moves to the other side of the roadway and then flows to the working face. In that scenario, in the range of 0~3 m from the working face, the air temperature decreases obviously, but the air temperature rises elsewhere, which can be seen in Figure 17. When the roadway section size is large, the airflow directly flows to the working face and then back to the exit of the roadway, the low temperature zone is very close to the working face and road wall. This implies that it is essential to control the movement of airflow, and the airflow should first cool down where people and machines are, which can promote the utilization of cool energy. As the roadway section size increases, the surface area of the surrounding rock of roadway increases, and the air temperature increases due to the increase in the heat release from the surrounding rock. The main region of temperature rise is in the Backflow zone of the right wall and Vortex zone. When the section area of roadway increases from 6.075 to 7.696 m, the air temperature at the exit of roadway rises from 27.9 to 30.1 °C, as shown in Figure 18. Thus, once the roadway section size increases during the construction of roadway, the cooling approaches should be strengthened correspondingly.

## 5. Orthogonal Test

The orthogonal test method is to select representative tests from a large number of tests according to the mathematical statistics and principle of orthogonality. It can not only greatly reduce the number of tests, but also can comprehensively analyze the experiment and find out the influence rule of each factor on the evaluation index of the experiment.

In this paper, a seven-factor and eighteen-level orthogonal (U_18_*(3^7^)) test is designed to evaluate the influence of factors such as the ventilation volume, initial temperature of ventilation airflow, advance rate of working face, distance between duct outlet and working face, roadway section size, temperature of surrounding rock and diameter of the air duct. The average temperature at the exit of the roadway (ATE) and the difference between the peak of air temperature and the air temperature in the exit of the roadway on the center line (DPE) is selected for the list analysis. The index of ATE can reflect the influence of the factors on the average air temperature in the roadway, and the DPE index depicts the degree of local heat accumulation. The orthogonal experiment table for the simulation is shown in Table 2. The maximum difference of test results for three groups of each influencing factor is defined as the Range, and the sensitivity of each factor on the roadway temperature field is determined by the Range, in other words, the greater the value, the larger the influence of the factor.

**Figure 19:**
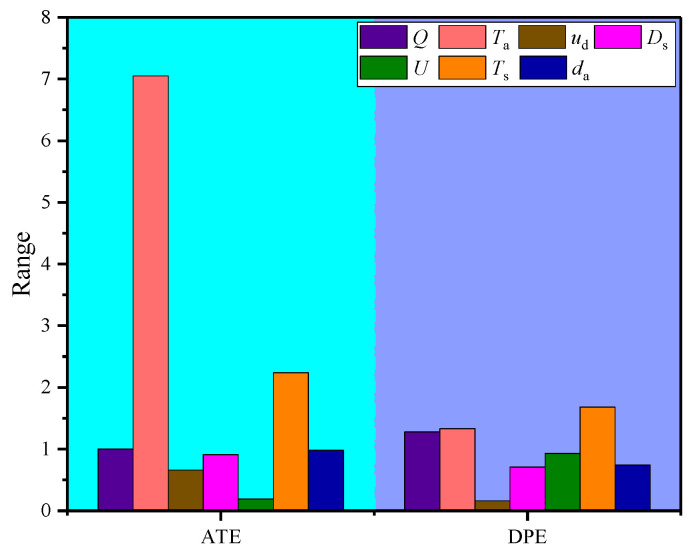
Sensitive comparison of the factors on the indexes of ATE and DPE.

Table 3 shows the sensitivity of factors for each index. The orthogonal test of the ATT presents that the Range of the initial temperature of ventilation airflow is the largest, indicating that the initial temperature of ventilation airflow has the greatest influence on the average air temperature in the roadway. The influence of the surrounding rock temperature is next in line for the average air temperature and the roadway section size is minimal. The factors impacting the average air temperature in the roadway in a descending sequence are as follows, the initial temperature of ventilation airflow > temperature of surrounding rock > ventilation volume > diameter of air duct > distance between duct outlet and working face > advance rate of working face > roadway section size. These factors have different performances on the local heat accumulation in the roadway. The temperature of the surrounding rock has the greatest influence on the DPE, which means that the higher the temperature of surrounding rock is, the easier it is to accumulate heat and form a local high-temperature zone in the roadway. The influence of the advance rate of working face to the local heat accumulation is the least. The sensitive comparison of the factors on the indexes of ATE and DPE is shown in Figure 19.

In general, in the construction of a roadway, the temperature of surrounding rock and initial temperature of ventilation airflow have the greatest influence on the average air temperature and local heat accumulation in the roadway. Therefore, reducing the temperature of surrounding rock or the initial temperature of ventilation airflow is the most powerful way to control the thermal hazard in the roadway. However, these two approaches are both costly for improving the cooling effect in practice. Increasing the ventilation volume is the most common method to promote thermal comfort, but there is a limit to adjust air temperature. Adjusting the size of the air duct and the distance between the duct and the working face are often neglected in the past, but in fact, it also can appropriately regulate the air temperature in the roadway, especially in local heat accumulation in the roadway.

## 6. Conclusions

The thermal stresses of the working environment in the roadway significantly affect worker health, labor productivity, and the failure likelihood of both equipment and workers. The thermal performance in an excavating mine roadway equipped with auxiliary ventilation was investigated by three-dimensional numerical simulations. The airflow pattern and temperature field in the excavating mine roadway under different ventilation times were obtained and analyzed. The critical factors, such as the ventilation volume, diameter of the air duct, distance between duct outlet and working face, advance rate of the working face and roadway section size, were investigated to determine how to change the thermal performance in the roadway. An orthogonal experiment was performed on the average temperature at the exit of the roadway and the difference between the peak of air temperature and the temperature of exit of the roadway on the center line, for examining the effect of different critical factors on the average air temperature and local heat accumulation in the roadway. The main conclusions are as follows:

(1) The airflow field is distinguished into three parts: Jet zone, Backflow zone, and Vortex zone. The triangular swirling vortex exists in the middle of the roadway and 4–5 m away from the working face where the heat is easily accumulated and the air temperature is high.
(2) Under the condition of continuous ventilation and excavation of roadway, the air temperature in the roadway decreases first and then go stabilized. The local high-temperature zone in the roadway moves forward with the advance of the working face.
(3) Increasing the ventilation volume can promote the thermal environment in the roadway, but there is a limit to adjust air temperature. Reducing the diameter of air duct or distance between the duct outlet and the working face will increase the airflow velocity near the working face and enhance the convective heat in the working face, which leads to an increase in air temperature in the roadway.
(4) The temperature of surrounding rock, the initial temperature of ventilation airflow and ventilation volume have a significant influence on the air temperature and local heat accumulation in the roadway, and decreasing the initial temperature of ventilation airflow and the temperature of surrounding rock is the key to control the heat hazard in an excavating roadway. The priority of control measures for heat hazard can be determined by referring to the sensitivity degree of factors.

## Figures and Tables

**Table 1:** Parameters used in the numerical simulation.

Parameters	Value
Density of rock, *ρ*_s_ (kg·m^−3^)	2600
Specific heat capacity of rock, *C*_ps_ (J·(kg·K)^−1^)	1300
Heat conduction coefficient of rock, *K*_ps_ (W·(m·K)^−1^)	3.5
Density of gas, *ρ*_g_ (kg·m^−3^)	1.213
Gas dynamic viscosity, *μ*_g_ (Pa·s)	1.84 × 10^−5^
Heat conduction coefficient of gas, *K*_pg_ (W·(m·K)^−1^)	0.259
Specific heat capacity of gas, *C*_pg_ (J·(kg·K)^−1^)	1012
The working face advance rate, *R*_a_ (m/h)	0.2
The diameter of air duct, *d*_a_ (m)	0.6
The distance between duct outlet and working face, *D*_s_ (m)	6
The initial rock temperature, *T*_r_ (°C)	45
The initial temperature of ventilation airflow, *T*_a_ (°C)	25

**Table 2:** U_18_*(3^7^) orthogonal experiment.

Test Number	Factors						
	Ventilation Volume *Q* (m^3^/min)	Initial Temperature of Ventilation Airflow *T_a_* (°C)	Advance Rate *u*_d_ (m/h)	Distance between Duct Outlet and Working Face *D*_s_ (m)	Roadway Section Size *U* (m^2^)	Temperature of Surrounding Rock *T_s_* (°C)	Diameter of Air Duct *d*_a_ (m)
1	1 (85)	1 (20)	1 (0.15)	1 (6)	1 (6.075)	1 (40)	1 (0.25)
2	1 (85)	2 (25)	2 (0.2)	2 (7)	2 (6.886)	2 (45)	2 (0.30)
3	1 (85)	3 (30)	3 (0.25)	3 (8)	3 (7.696)	3 (50)	3 (0.35)
4	2 (102)	1 (20)	1 (0.15)	2 (7)	2 (6.886)	3 (50)	3 (0.35)
5	2 (102)	2 (25)	2 (0.2)	3 (8)	3 (7.696)	1 (40)	1 (0.25)
6	2 (102)	3 (30)	3 (0.25)	1 (6)	1(6.075)	2 (45)	2 (0.30)
7	3 (119)	1 (20)	2 (0.2)	1 (6)	3 (7.696)	2 (45)	3 (0.35)
8	3 (119)	2 (25)	3 (0.25)	2 (7)	1(6.075)	3 (50)	1 (0.25)
9	3 (119)	3 (30)	1 (0.15)	3 (8)	2 (6.886)	1 (40)	2 (0.30)
10	1 (85)	1 (20)	3 (0.25)	3 (8)	2 (6.886)	2 (45)	1 (0.25)
11	1 (85)	2 (25)	1 (0.15)	1 (6)	3(7.696)	3 (50)	2 (0.30)
12	1 (85)	3 (30)	2 (0.2)	2 (7)	1(6.075)	1 (40)	3 (0.35)
13	2 (102)	1 (20)	2 (0.2)	3 (8)	1 (6.075)	3 (50)	2 (0.30)
14	2 (102)	2 (25)	3 (0.25)	1 (6)	2 (6.886)	1 (40)	3 (0.35)
15	2 (102)	3 (30)	1 (0.15)	2 (7)	3 (7.696)	2 (45)	1 (0.25)
16	3 (119)	1 (20)	3 (0.25)	2 (7)	3(7.696)	1 (40)	2 (0.30)
17	3 (119)	2 (25)	1 (0.15)	3 (8)	1(6.075)	2 (45)	3 (0.35)
18	3 (119)	3 (30)	2 (0.2)	1 (6)	2 (6.886)	3 (50)	1 (0.25)

**Table 3:** Orthogonal test Range calculation of air temperature.

Factors	Ventilation Volume		Initial Temperature of Ventilation Airflow		Advance Rate		Distance between Duct Outlet and Working Face		Roadway Section Size		Temperature of Surrounding Rock		Diameter of Air Duct	
Index	ATE	DPE	ATE	DPE	ATE	DPE	ATE	DPE	ATE	DPE	ATE	DPE	ATE	DPE
Level 1	30.78	2.18	26.65	3.10	30.01	2.38	30.83	1.87	30.25	2.73	29.40	1.60	30.69	1.88
Level 2	29.78	3.00	30.65	2.03	30.67	2.30	29.92	2.45	30.30	2.37	29.95	2.02	30.58	2.40
Level 3	30.43	1.72	33.70	1.77	30.31	2.22	30.25	2.58	30.44	1.80	31.64	3.28	29.71	2.62
Range	1.00	1.28	7.05	1.33	0.66	0.16	0.91	0.71	0.19	0.93	2.24	1.68	0.98	0.74
Rank	3	3	1	2	6	7	5	6	7	4	2	1	4	5

## Data Availability

Not applicable.
